# Miasm

**Published:** 1879-01

**Authors:** 


"MIASM,"

From a Greek word which means *emanation;* that is, ARISING FROM; because it comes up from the surface of the earth. It is a short word, but it brings weary sickness and agonizing death to hundreds of thousands every year; it will bring sickness and death, sooner or later, to many a reader of this article; but a sickness and death which could have been avoided.

MIASM is the principal cause of nearly every "epidemic" disease; that is, of every sickness which "falls upon the people;" attacking numbers in any community, such as fever and ague, diarrhea, dysentery, cholera, bilious, intermittent, congestive and yellow fevers. But it is gratifying to know that it is an avoidable cause of disease. Money and wisely directed efforts can banish it from almost any locality. All that is needed is to know the laws of miasm, and wisely adapt ourselves to them.

In 1860, one of the daily papers of New-Orleans stated: "The yellow fever has broken out in the city under every conceivable variety of circumstances; when the streets were clean and when they were filthy; when the river was high and when it was low; after a prolonged drought, and in the midst of daily torrents; when the heat was excessive, and when the air was spring-like and pleasant; when excavations and disturbances of the soil had been frequent, and when scarcely a pavement had been laid or a building erected. Almost the only fixed and undeniable fact connected with the disease is, that its prevalence is simultaneous with the heats of summer, and that frost is its deadly enemy." Here, then are two important laws of miasm; and scientific observation directed to that special point in all countries, confirms the two great truths, that,

First. Miasm prevails in hot weather.

Second. Miasm can not exist as a cause of disease in cold weather.

Third. An inference is drawn embodying a third law of miasm, which is, that it is a cause of disease only from June to October, in our latitudes.

Fourth. A fourth law of miasm is confirmed by the now historical fact, that for three summers yellow fever was not known as an epidemic in New-Orleans; because, from the scientific views held by those in power in that city in the early summer of 1861.

It is within the memory of the present generation, that some thirty years ago or more, the city of Louisville, in Kentucky, was one of the most pestilential spots in the habitable West. But by a wise system of filling and draining, it is now one of the healthiest, as well as one of the most beautiful cities of the great valley.

We have then arrived at four controlling facts in reference to miasm—that heat and moisture are essential to its production in any locality; that it can not exist where there is severe frost or great dryness.

But as it is known the world over that miasm never exists in deserts, where there is nothing but dry sand and a burning heat, it is clear that something more than heat is necessary to cause miasm.

is known that vigorous growing bushes, or hedges or trees, between a miasm-producing locality and a dwelling, antagonize the miasmatic influences; the living leaves seeming to absorb and feed upon the miasm; but there should be a space of fifty yards at least between the hedge and the house; and the thicker and broader and higher the hedge, the better; and the nearer the leaves are to the ground the better, for the miasm gropes on the surface in its greatest malignity; and is seldom concentrated enough at the hight of ten feet to be materially hurtful to man, unless it comes up a slope Hence, in the old cities of the world, in the times of plagues and pestilences, the people who could not "go to the country," had a custom among them to live in the upper stories of their dwellings, while the sickness raged; they would not even come down stairs to obtain marketing, but would let down baskets by ropes to the country people, for the provisions they had to sell. But they failed to discover why the country people could come to town with impunity, while they themselves were safe from disease in proportion as they lived in the upper stories of their dwellings. But a law of miasm has since been determined which beautifully unravels the mystery. Miasm is condensed by cold, made heavy, and falls to the earth, hovering, as it were, within a foot of its surface; hence is not breathed, unless a man sleeps on the ground. On the other hand, heat so rarefies miasm, as to make it comparatively innocuous. Hence the coolness of the early morning and of sundown threw the miasm to the surface, by condensing or concentrating it, and thus making it heavy; while the heat of the day, of a summer's sun, so rarefied and lightened the miasm, as to send it upward to the clouds. *The country people came to town in the daytime!*

Less than fifty years ago, the yellow fever and other deadly diseases prevailed in Charleston, South-Carolina, and it was known to be certain death, except to the very hardy or the acclimated, to sleep in the city a single night. Yet the merchants came to town at mid-day, under a blistering July sun, with perfect impunity. Hence, from June to October, it is best for farmers' families to sleep in the upper stories of their dwellings. In this connection, it is practically useful to know that the most malignant agencies of nature may be rendered harmless by a little observation, and the wise use of a little knowledge. Miasm is most pernicious about sunset and sunrise, because the cooling of the atmosphere at the close of the day causes it to become condensed above, to become heavy and fall to the earth, where it is breathed; while after sundown, it has settled so near the earth as to be below the mouth and nostrils, hence it is not breathed. When the sun begins to rise in the morning, the miasm begins to warm and to ascend, but after breakfast it is so high as to be above the point at which it can be breathed; and besides, it is so rarefied, so attenuated, as to be innocuous. Therefore, the great practical truth beautifully follows, that miasm exerts its most baleful influence on human health about sunrise and sunset; hence, of all the hours of the twenty-four, these are the most hurtful, in which to be out of doors; and for the same reason, the hours of midday and midnight are the most healthful to be in the open air in miasmatic seasons and countries; that is, from June to October, north of the thirty-fifth degree of north latitude.

But unfortunately the cool of the early morning and the late afternoon are the most pleasant times in the twenty-four hours for field work, and the industrious farmer will be exceedingly loth to spend these hours in-doors, should his house be already located in a miasmatic situation. There is, however, an almost infallible preventive of any ill effects arising from such an exposure to miasm about sunrise and sunset, and one that is easy of practical application under almost any ordinary circumstances; and it ought to be made known and repeated millions of times through the public prints every year, until the information has reached every farmer's dwelling throughout the United States. Farmers, whose houses are already built in malarial districts, such as in low "made" lands, near ponds and stagnant water, or in the neighborhood of sluggish streams or marshy places, may exempt themselves almost altogether from the whole class of malarial diseases, such as diarrheas, dysenteries, chills and fevers of nearly every grade, by eating a hearty and warm breakfast before they put their heads out of doors in the morning, and by taking their suppers just before sundown; the philosophy of the matter is, that a hot or hearty meal so excites the circulation, and so invigorates the whole frame, that it acquires the power of resisting the disease-engendering influences of miasm. A neglect of such a simple precaution, in certain districts where malaria is known to exist in a concentrated form, is a cause of death so common as to be known and guarded against by the most uneducated laborers. A gentleman, a native of the city of Rome, informed the writer that multitudes of agricultural laborers who have been employed during the day in the low, level damp fields near the city, come into town about sundown and sleep in the streets and on the steps and stoops of houses, in order to avoid the sickly atmosphere of the evening in the "marches." No less a personage than a young king lost his life within two years, under the following circumstances: Having to pass the night in one of his journeys at a house located in the midst of an extensive low land or marsh, and wishing to be on horseback early in the morning for a hunt, the landlord pressed upon him the danger of being out early, and that at least he should take his breakfast first. The impatient youth was observed early next morning sitting at his open window, enjoying, as he thought, the delightful air as it blew in upon him, and soon after ordered his horses. He became ill and died of fever in a few days. The writer has lived among the Creoles of Louisiana where vegetation is rank in swamps, upon which the hot summer's sun beams with fiery power for many hours every day; but they are proverbially exempt from fevers, as are Northerners also, who adopt the habits of the Creoles—that is, to have their breakfast, or at least a cup of hot strong coffee with milk, brought to their bedsides *before they get up* of a morning. The value of this practice is known and appreciated all over the South; so that while it is greatly better to locate a house where miasm can not reach it from ponds or sluggish streams or bottom lands, a farmer whose house is already thus situated, is not without an efficient remedy in the plan proposed above.

But there is another infallible remedy against miasmatic diseases as to families who feel themselves compelled to live in a house exposed to miasm. It was stated awhile ago that heat so rarefied miasm as to render it innocuous. No family can be troubled with fever and ague in any ordinary locality, if from June to October a brisk fire is kindled in the family-room, to burn for an hour about sunrise and sunset, and if the family are required to repair to that room morning and evening and remain there, at least until they get their breakfast in the morning, and their supper at the close of the day.

It follows then that ordinarily, there is nothing unhealthful in the night-air after supper; on the contrary, health would be promoted, and important social benefits would accrue to country neighborhoods, if two or three nights of every week, after tea, were spent in friendly visiting, remaining not later than ten; thus encouraging that interchange of social associations which diffuses intelligence, promotes kindly feeling, enlarges the views, expands the ideas, and elevates the whole character, by cultivating the tastes as to dress, tidiness of person, and the imitation or copying after any ornament or improvement of the grounds and dwellings of the neighborhood. In this way, one intelligent practical farmer in a neighborhood, by occupying a house which he has built or remodeled for himself, so as to have all the comforts and conveniences which knowledge and observation and experiment have found to contribute largely to the health, happiness, and thrift of the occupants, will prove a leaven which shall spread from one habitation to another in a comparatively short time, until every dwelling in the circuit of many miles will be more or less improved, and thus the face of the whole country be changed for the better, with the promise and realization of a further progress, onward and upward.

### RECAPITULATION.

Although the statements which have been made were presented in connection with the selection of the most healthful locality for building a new family residence, they are practically applicable to all cases wherein it may be desirable to make a house already built more comfortable and more healthful than it is, because, from what has been stated, it will be seen that a dwelling already erected should not be hastily and blindly abandoned merely on account of its insalubrity, for in the light of the above statements, it may be found that the causes of any present sickness are of a transient or of a remediable character, which may thus be illustrated:

The most favorable circumstances for the production of a miasmatic epidemic, speedy, malignant, and wide-spreading, are the exposure of the muddy bottom of a pond or sluggish stream to the beaming heat of a summer's sun. In less than a week whole neighborhoods have been stricken with disease, yet under such circumstances, and according to the well-established laws of miasm, five families may dwell within half a mile of a drained mill-pond, and yet only one will suffer from it, while the other four will remain exempt from unusual disease:

First. If a rapid stream half a mile wide runs between the drained pond and the house.

Second. If there is interposed a thick hedge or growth of living luxuriant trees or bushes. A treble row of sun-flowers are known to have answered the purpose in repeated cases.

Third. If the prevailing winds from June to October are from the house toward the pond.

Fourth. If the house be on a steep hill.

The reasons for the above exemptions are here shortly recapitulated:

First. Miasm does not cross a wide, rapid stream.

Second. Miasm is absorbed by thick, living luxuriant foliage.

Third. Miasm can not travel against the wind.

Fourth. Miasm can not ascend a high, steep hill.

There is no mystery in these variations, nor any complexity, when the laws of miasm are thoroughly understood.

It will be practically useful for the young farmer, in a pecuniary point of view, to understand further that in one year a house on the banks of a mill-pond or sluggish stream may be visited with sickness; the very next year that same house may be exempt, because it is a very cold summer; the third year it will escape, because it is a very hot summer; the fourth year it will be a very healthful habitation, because it has been a very wet summer. Why these variations?

First. Miasm can not form, or if it does, can not rise through a foot or two of depth of water, and the wet summer kept the bed of the pond covered.

Second. The hot summer dried the bed of the pond to dust, and there can be no miasm without dampness.

Third. The cold summer did not give the degree of heat necessary to the generation of miasm—that is, eighty degrees of Fahrenheit.

These principles fully explain the apparent mystery of the epidemics in New-Orleans, already referred to in the first part of this paper.

An illustration of the laws of miasm, which the reader will never forget, was had during a cholera summer in Boston, under the following circumstances: the city authorities inaugurated a most perfect system of cleanliness. Efforts were made to procure the services of the most reliable men to visit every house from cellar to garret, and compel the removal of every thing which could have even a remote tendency to invite the fearful scourge. The results were admirable; there was not a single case of cholera except in a very restricted district—in fact one family only was attacked. A more special examination was instituted, when there was found in a remote corner of the cellar a large pile of the accumulations of bad housekeeping for years; and this was in a state of putridity. On its removal, and the plentiful use of the most powerful disinfectants, the disease at once disappeared and did not return!

